# Comment on Nejati et al. Spatiotemporal Patterns and Historical Overview of *Aedes* Mosquitoes in Iran: A Systematic Review. *Trop. Med. Infect. Dis.* 2026, *11*, 131

**DOI:** 10.3390/tropicalmed11080236

**Published:** 2026-08-21

**Authors:** Tahereh Sadat Asgarian, Mohammad Mehdi Sedaghat

**Affiliations:** Department of Vector Biology and Control of Diseases, School of Public Health, Tehran University of Medical Sciences, Tehran 14176-13151, Iran; t.s.asgarian@gmail.com

## 1. Introduction

In this paper, we critically review the article entitled “Spatiotemporal Patterns and Historical Overview of *Aedes* Mosquitoes in Iran: A Systematic Review” published in *Tropical Medicine and Infectious Disease* (2026, 11, 131) [1]. The authors provide a comprehensive assessment of the spatial and temporal distribution of *Aedes* species in Iran, highlighting recent invasion events and discussing their implications for public health preparedness. We congratulate the authors on this valuable contribution to the field and would like to offer several comments and additional perspectives that may further strengthen the interpretation and future application of their findings.

## 2. Comments

Several points in this interesting article merit further consideration, and we would like to share our reflections in this letter.

In the introduction, the authors state that 15 species of the genus *Aedes* occur in Iran. However, Table 1 lists only 14 species. While we recognize the taxonomic complexity regarding *Ae. asiaticus*—specifically its recent elevation from subspecies status and the historical difficulty in distinguishing it from *Ae. pulcritarsis*—its omission from the list creates a discrepancy with the authors’ own introductory claim of 15 species. Given its current recognition as a valid species in the Iranian fauna [2], its inclusion in Table 1 would have provided greater taxonomic clarity. We maintain that for the sake of taxonomic integrity and to prevent reader confusion, all validly recognized species within the review’s scope should be explicitly listed in Table 1.

The review appears to have excluded governmental and technical reports, including those published by the Iran Center for Disease Control (ICDC), which may have contained valuable distribution records for *Aedes* species. For instance, recent reports indicate the presence of *Ae. aegypti* in Fars Province [3,4] and *Ae. albopictus* in Zanjan [3,5] and Qazvin [3,6] Provinces. The exclusion of such valuable data sources may have led to an incomplete picture of the current distribution of these invasive species. For example, the article presents these provinces (Fars, Zanjan, and Qazvin) as lacking *Aedes* records, which directly contradicts the aforementioned ICDC reports. A systematic review should not be limited to indexed articles alone, especially in the case of vectors of diseases and invasive *Aedes*, where governmental reports (the gray literature) are far more crucial than scattered published papers.

Additionally, Keshavarzi et al. (2017) reported the presence of *Ae. caspius* and *Ae. vexans* in Fars Province [7]; however, this study was not included in the review.

Zaim (1987) reported the presence of *Aedes* mosquitoes (including *Aedes caspius*) in several provinces (Razavi Khorasan, Yazd, and Zanjan) [8] during the same time period covered by the authors, but these records are not reflected in their findings. It is noteworthy that the authors cite Zaim (1987) [8]; however, in Figure 2 these provinces lack reports of *Aedes* species.

In Section 3.2.2 regarding Kurdistan Province, there appears to be an inconsistency between the textual description and Figure 2. The text notes that *Aedes* specimens were not detected “in several surveys,” which may imply specific localized findings rather than a provincial absence. However, Figure 2 indicates the presence of two *Aedes* species within this province. To resolve this apparent discrepancy, we recommend that the authors provide specific citations for the mentioned surveys.

While we acknowledge the authors’ use of the qualifier “in several surveys”, the phrasing remains problematic for a systematic review. By highlighting that these regions “yielded no specimens” in certain surveys, the text implicitly suggests a lower prevalence or a lack of presence in mountainous areas, which creates a cognitive dissonance for the reader when contrasted with Figure 2.

The statement claiming that “In Qom, exceptionally high adult densities were recorded in 2025” is not accompanied by any supporting reference and justification. We kindly request the authors to provide the source for this data, as it represents an important epidemiological observation that needs verification.

Lorestan Province also appears to be lacking records of *Aedes* mosquitoes, despite a report by Kayedi et al. (2020) that identified *Ae. vexans* in this area for the first time [9]. This inconsistency indicates that some important details may have been missed during the review process. A thorough review of the complete texts of the available research, instead of relying only on abstracts, could have potentially clarified these discrepancies and offered a more accurate understanding of *Aedes* mosquito distribution in Iran.

## 3. Conclusions

While there are notable concerns, the systematic review conducted by Nejati et al. provides a valuable foundation for understanding the distribution of *Aedes* species in Iran. However, addressing the inconsistencies mentioned, specifically the omission of Ae. asiaticus in Table 1, the discrepancies between the text and figures, and the need for more comprehensive inclusion of the gray literature, is crucial. Such refinements are not merely academic; they are vital for public health. Accurate taxonomic identification and precise spatiotemporal mapping are fundamental to designing effective vector control programs, optimizing resource allocation, and anticipating the emergence of diseases like Dengue and Zika in vulnerable regions. Ensuring the reliability of these records will directly enhance the ability of public health authorities to implement data-driven interventions. We trust that the authors will consider these comments constructively and, if warranted, issue a corrigendum to correct these oversights.

## Data Availability

No new data were created or analyzed in this study. Data sharing is not applicable to this article.
